# The White Feather in Evidence

**Published:** 1909-04

**Authors:** 


### EDITORIAL DEPARTMENT.

### THE WHITE FEATHER IN EVIDENCE.

GALVESTON, TEXAS, April 2, 1909.

*Dr. F. E. Daniel, Austin, Texas.*

DEAR SIR: Enclosed please find a letter addressed by me to Dr. I. C. Chase, editor of the *Texas State Journal of Medicine*, February 18, 1909; also, please find letter from him to me and copy of my letter to him of this date.

If you feel so inclined, I would be glad to have you publish it in this month's issue of your JOURNAL.

Your most respectfully and fraternally,

A. W. FLY, M. D.

---

STATE MEDICAL ASSOCIATION OF TEXAS
Publishers of the
TEXAS STATE JOURNAL OF MEDICINE.
I. C. Chase, Secretary and Editor-in-Chief,
Room 502 Continental Bank Building.

FORT WORTH, TEXAS, March 26, 1909.

*Dr. A. W. Fly, Galveston, Texas.*

DEAR DOCTOR: Replying to your of the 24th, I beg to say that the submission to the Trustees of the communication sent me [February 18th] has not yet been completed. As soon as the details of the disposition of this article have been settled, I will communicate with you.

Respectfully yours,

I. C. CHASE.

[Note the date of Dr. Fly's letter, February 18th, and Dr. Chase's, March 26th, about six weeks. On receipt of the latter Dr. Fly wrote again saying that he had positive information to the contrary and that the paper had been refused publication March 3rd. Name of informant on request. The controlled editor and the collar clique dared not let the blindly led members know the truth.]

---

GALVESTON, TEXAS, April 2, 1909.

*Dr. I. C. Chase, Secretary Texas State Journal of Medicine, Fort Worth, Texas:*

Yours of March 26, 1909, was received, and evasive contents carefully noted.

It is now plain that you do not intend publishing my paper, because you have had from February 23, 1909, to April 1, 1909, to hear from all parties who have any connection with the *Journal.*

I realize that you, through your autocratic board, have a right to publish or reject any matter that may not accord with your views, but you have not the right to be unjust or unfair to a member of the profession who has practiced ethics, and not written or talked it, for more than thirty years.

It is very plain the reason why your adroit avoidance of stating to me frankly the objection to the publishing of this letter. You seem to be afraid that some other journal, or possibly some independent paper, might draw the attention of the profession to these matters prior to the meeting of the Association. This is exactly why I wanted you to publish it in the March *Journal.* Your letter to me dated February 23d—the date that you received my letter, stating that no matter could be used after the 18th, seemed a reasonable excuse, but forty days have elapsed since. You owe it to me, you owe it to the profession, not less than yourself, to deal frankly and fairly upon all matters pertaining to ethics. The questions that I propounded to you are questions that are of mutual interest to every man who practices honest medicine.

It is a matter of information to many of the members of the profession that I have no respect whatever for the present hermaphrodite or mixed Board of Medical Examiners. I have nothing personal against, or any criticism to make of the personnel of such Board. They may be the finest gentlemen in the land, but the mere fact that it is composed partly of regulars, homeopathists, eclectics, and osteopaths (and magnetic healers, Christian Scientists and veterinary surgeons are excluded) shows to me a partisan spirit that seems to justify some of the criticisms that have heretofore been made.

Feeling that your failure to state to me the exact attitude of the Board and yourself upon this letter written you, is unfair and almost bordering upon the hypocritical, I hereby demand that you at once return my manuscript or answer to me personally when I see you for such disregard of the rules of common courtesy.

A. W. Fly, M. D.

---

Here follows the letter referred to, and the publication of which, *before the May meeting,* was shirked—for obvious reasons; couldn't face the music. Is the *Journal of Medicine* a close corporation run in the interest of The Octopus and "McCormack, the pardoned?"—Daniel.

GALVESTON, TEXAS, February 18, 1909.

*Dr. I. C. Chase, Secretary T. S. M. A.; Editor Texas State Journal of Medicine, Fort Worth, Texas:*

Assuming that the official organ of the Texas State Medical Association is published in the interest of the regular medical profession of Texas, and, is, therefore, an open forum for the discussion of matters of interest and importance to the membership of the Association, I ask you, in no spirit of controversy, but in all fairness, to publish this letter, and your reply in the March issue of their *Journal.* I desire the information for myself and for the profession at large. They are entitled to it in order that they may govern themselves accordingly. These questions are asked me, and I refer them to you, as the official expounder of the policy governing our organization. You, Dr. Chase, being a Northern man, may not attach the importance to one phase of the subject that Southern gentlemen do, as you are naturally not expected to look at it from their viewpoint. The Southern physicians will never affiliate with the negro; you may be sure of that, nor will the true physician bred in the school of legitimate regular medicine ever affiliate with the irregulars whom you denominate the "Minor Schools," despite the attempt to force this affiliation by recognition and fellowship on that monstrosity—the Mixed Board. You express surprise and regret that there are "over 3600 physicians in Texas practicing regular medicine who are, or should be, eligible to membership in their county societies" (and, therefore, in the State Association), and urge them to join.—*Texas State Journal of Medicine,* January, 1909, page 216. Your appeal "To Nonmembers of County Societies" (*loc cit.*) is not addressed solely to the 3600 "practicing regular medicine," but to *all* who are not members; for, you say, "Your name appears among 4050 others, as physisicans who are not affiliated with the organized medical profession of the State." "Of this number approximately 175 are Eclectics, 105 are Homeopaths, 30 Physio-Medicals, and 70 Osteopaths." Those aggregate 380. Deduct this from 4050, and we have 3670 "physicians in Texas practicing regular medicine," instead of "over 3600 not members of our organization." It is to be presumed that this discrepancy of 70 are negroes, for you are well aware that there are many negro physicians licensed and registered, and "verified," who are "practicing regular medicine in Texas," and who are, according to your statement, "eligible to membership." (There are probably double or treble that number, and almost without exception they are "regular." I

never heard of one of the "Minor School" persuasion.) You say (*loc cit.*) "The new law requiring registration * * * has enabled us for the first time to determine how many and who are licensed physicians." It is therefore evident that you must have ascertained "how many and who" of the negro physicians are registered and eligible. Why, then, did you not give the number? Why do you discriminate against them alone, when you include all others, and they, too, being (assumed) of the "regular" school? Let's have it. The members of the State Medical Association already (under the McCormack plan), being *disfranchised,* 95 per cent of them having no vote or voice in any matters pertaining to the interests and welfare of the regular profession, wish to know and have a right to know:

1. Are negro physicians eligible to membership in the State Association?

2. If not, why not?

3. Do we understand that you appeal to the 380 mixed physicians outside of the fold to come in? Are Osteopaths, Homeopaths, Eclectics, Physio-Medicals *et id. om. gen.* eligible to membership? If not, what does your appeal mean? If these questions are answered in the affirmative (and I see no escape from that construction), shall we soon have in our Association a Section on Homeopathy, a Section on Osteopathy, a Section on Eclecticism, a Section on Physio-Medicine? And as every city where we meet entertains the State Medical Association with ball, banquet and receptions at homes of leading citizens, shall this mixed element be invited? If not, why not?

You express surprise that of the 4050 (outs) plus 3322 (ins)—7372 "physicians in Texas duly licensed"—only 3322 are members of the State Association. That is 45 per cent of the estimated 7372 (by you). Of the 3322 (ins) plus 3600 (outs)—6922 *regulars* (including the registered negroes, of course)—there are 47 per cent (3322) members; less than one-half of the regular profession. (By the bye, I recall a statement you published editorially some two or three years ago that "about 90 per cent of the reputable physicians of Texas had already enrolled." Has there been, then, so great a falling off, supposing both your statements to be true? Or supposing that in the enthusiasm of your new honors and dignity in those earlier days, you made the reckless statement referred to (guessed at it), and that there are, as you now show, only 47 per cent of the regular (reputable?) physicians enrolled. Why, why, do they not enroll? Do you know a reason?

I could give you many reasons. Shall I give you one, two, three? The fear of beng denounced as a "scab and not in good standing"; of being left out of Dr. Simmon's Register; of having insurance companies told that an applicant for medical examiner is not a member of the Association. These coerce many to join. and to "pay for the *State Journal of Medicine,* whether they want it or not." (These things are freely written to me.) But, on the other hand, there are many (53 per cent, more than half of the regular profession) who *stay out,* not caring whether they are scabs or not, or whether they are in the Directory or not; and a very large number of those enrolled never attend county or State meetings; they simply *stay away.* There is noticeable at each annual meeting the absence of most of the older and former leaders. There is widespread dissatisfaction with the abolition of the annual volume of transactions; with having the hands of the great majority tied; with the denunciation of those who claim the right to prescribe certain proprietary medicines, which, for years, many, a great many of reputable physicians (you, Dr. Chase, amongst them, perhaps), have used and found useful, and never thought it was wrong, until told so by the party now in control of the great machine at Chicago; with the denunciation as unclean and infectious and unworthy of support, of the three Texas medical journals, the old friends of former days, and that, too, by you and other editors of controlled organs, which, at the time it was written, were carrying some of the very advertisements so vehemently denounced. It is only since December that the *Journal* of the Association has dropped them. (See editorial in January number, page 216.)

Respectfully,

(Signed) A. W. Fly, M. D.

If you refuse to publish this, kindly return it, giving your reasons.

[He neither published it nor gave any reasons for refusing to do so; and instead of returning the MSS., *kept it,* and sent Dr. Fly a copy.—Dr. Daniel.]

---

Dr. J. S. Price, Beaumont, writes and says: "I have been with the Red Back twenty years, and hope to see it flourish, with its wise and genial editor, twenty years more." He also sends us a new subscriber Every fellow ought to do likewise.

